# Cross‐talk between motor neurons and myotubes via endogenously secreted neural and muscular growth factors

**DOI:** 10.14814/phy2.14791

**Published:** 2021-05-01

**Authors:** Jasdeep Saini, Alessandro Faroni, Adam J. Reid, Vincent Mouly, Gillian Butler‐Browne, Adam P. Lightfoot, Jamie S. McPhee, Hans Degens, Nasser Al‐Shanti

**Affiliations:** ^1^ Musculoskeletal Science & Sports Medicine Research Centre Department of Life Sciences Manchester Metropolitan University Manchester UK; ^2^ Blond McIndoe Laboratories Division of Cell Matrix Biology and Regenerative Medicine School of Biological Sciences Faculty of Biology Medicine and Health University of Manchester Manchester Academic Health Science Centre Manchester UK; ^3^ Dept. of Plastic Surgery & Burns Wythenshawe Hospital Manchester University NHS Foundation Trust Manchester Academic Health Science Centre Manchester UK; ^4^ Center for Research in Myology Sorbonne Université‐INSERM Paris France; ^5^ Department of Sport and Exercise Sciences Manchester Metropolitan University Manchester UK; ^6^ Lithuanian Sports University Institute of Sport Science and Innovations Kaunas Lithuania

**Keywords:** cross‐talk, motor neurons, muscle, neural growth factors, neuromuscular junction (NMJ)

## Abstract

Neuromuscular junction (NMJ) research is vital to advance the understanding of neuromuscular patho‐physiology and development of novel therapies for diseases associated with NM dysfunction. *In vivo*, the micro‐environment surrounding the NMJ has a significant impact on NMJ formation and maintenance via neurotrophic and differentiation factors that are secreted as a result of cross‐talk between muscle fibers and motor neurons. Recently we showed the formation of functional NMJs *in vitro* in a co‐culture of immortalized human myoblasts and motor neurons from rat‐embryo spinal‐cord explants, using a culture medium free from serum and neurotrophic or growth factors. The aim of this study was to assess how functional NMJs were established in this co‐culture devoid of exogenous neural growth factors. To investigate this, an ELISA‐based microarray was used to compare the composition of soluble endogenously secreted growth factors in this co‐culture with an a‐neural muscle culture. The levels of seven neurotrophic factors brain‐derived neurotrophic factor (BDNF), glial‐cell‐line‐derived neurotrophic factor (GDNF), insulin‐like growth factor‐binding protein‐3 (IGFBP‐3), insulin‐like growth factor‐1 (IGF‐1), neurotrophin‐3 (NT‐3), neurotrophin‐4 (NT‐4), and vascular endothelial growth factor (VEGF) were higher (*p* < 0.05) in the supernatant of NMJ culture compared to those in the supernatant of the a‐neural muscle culture. This indicates that the cross‐talk between muscle and motor neurons promotes the secretion of soluble growth factors contributing to the local microenvironment thereby providing a favourable regenerative niche for NMJs formation and maturation.

## Graphical abstract

Formation of neuromuscular junction between motor neuron and myoblast.

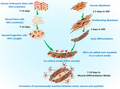

## 1 INTRODUCTION

Neuromuscular Junctions (NMJs) are the highly specialized peripheral synapses that translate neural signals received from motor neurons to contractile activity in skeletal muscle cells (Witzemann, 2006). During embryological myogenesis cross‐talk between muscle fibers and motor neurons appears indispensable for the development and maintenance of the neuromuscular system (Cisterna et al., 2014). The significance of this cross‐talk is also reflected by the limited differentiation *in vitro* of a‐neurally cultured skeletal muscle cells to non‐contracting myotubes (Delaporte et al., 1986) that contrasts with highly differentiated contracting myotubes in co‐cultured skeletal muscle cells and motor neurons (Saini et al., 2019). The interdependence of these tissues is further apparent when considering neurodegenerative disorders where damage or pathologies of peripheral nerves is associated with significant muscle wasting and degeneration (Tintignac et al., 2015). These observations indicate that particularly muscle cell differentiation is dependent not only on functional innervation, but also on the cross‐talk between muscle cells and the nervous system. This could be orchestrated via the secretion of critical trophic factors released into their microenvironment.

Skeletal muscle cells are an important source of growth factors, cytokines and neurotrophins. They also express many receptors for these growth factors, suggesting that neurotrophic signalling plays an important role in skeletal muscle cell development and innervation (Chevrel et al., 2006; Gonzalez et al., 1999; Griesbeck et al., 1995; Sakuma & Yamaguchi, 2011). Indeed, neurotrophin‐4 (NT‐4) and neurotrophin‐5 (NT‐5) null mice display clear defects in muscle development and function, indicating the significance of NT‐4/5 in skeletal muscle fiber differentiation (Carrasco & English, 2003). Additionally, neurotrophin‐3 (NT‐3) has been implicated in the formation of muscle spindles (Ernfors et al., 1994) and some dystrophic muscle pathologies have been linked with altered nerve growth factor (Capsoni et al., 2000). The discovery of BDNF receptor expression in skeletal muscle cells and muscle satellite cells has opened up interest to its postulated role in skeletal muscle cell development and regeneration (Chevrel et al., 2006). A study investigating the role of BDNF identified its supportive function in MN growth, survival, differentiation, regeneration and synaptic differentiation (Lee & Jun, 2019) while another study demonstrated the importance of BDNF.

For normal myogenic differentiation and regeneration following injury (Kolarow et al., 2007). Interestingly, in skeletal muscle cells BDNF knockout was associated with increased myoblast differentiation and the maintenance of the satellite cell population (Mousavi & Jasmin, 2006). This may explain why an increase in BDNF expression has been observed following various peripheral nerve injuries denoting its role in stimulating satellite cells in muscle damage (Omura et al., 2005). Likewise findings from BDNF null and muscle specific BDNF KO mice studies exhibited inhibition of myogenic differentiation and regeneration (Clow & Jasmin, 2010). BDNF signalling has also been shown to promote the withdrawal of weaker motor neuron contacts whilst leaving active motor neuron terminals intact, thereby regulating synaptic density in synaptogenesis (Garcia et al., 2010). Furthermore, the smaller muscles from diabetic than normal mice show a lower expression of NT‐3 and NGF mRNA and a higher expression of BDNF mRNA (Fernyhough et al., 1995, 1996, 1998; Fernyhough, Diemel, Hardy, et al., 1995; Ihara et al., 1996).

The glial‐cell‐line‐derived neurotrophic factor (GDNF), first discovered in glial cells, plays a role in the support of central nervous system dopaminergic neurons (Lin et al., 1993). It has been shown that overexpression of GDNF in skeletal muscle cell induces hyper innervation through increased sprouting (Nguyen et al., 1998), which corresponds with the significant transient expression of GDNF at NMJs during embryonic myogenesis *in vivo*. Given these observations, it has been suggested that GDNF may help maintain cholinergic motor neurons throughout aging (Ulfhake et al., 2000) and the increased GDNF expression in denervated human skeletal muscle cells (Lie & Weis, 1998) may be part of an attempt to restore innervation.

Vascular endothelial growth factor (VEGF) is not only an endothelial mitogenic factor, but also plays an important role in maintenance of the motor neuron. Therefore, it is considered a promising therapeutic agent for amyotrophic lateral sclerosis and other neurodegenerative diseases (Tovar et al., 2014).

As discussed above, endogenous growth factors and neurotrophins are vital for survival, development, plasticity and function of neurons, muscle and NMJ *in vivo* (Huang & Reichardt, 2001). Therefore, it is not surprising that most muscle‐nerve co‐cultures introduce exogenous growth factors (Das et al., 2007; Guo et al., 2011, 2014; Puttonen et al., 2015; Rumsey et al., 2010). While this enhances differentiation, it at the same time complicates drug discovery and toxicology studies due to possible interactions of the compounds being screened with factors contained within the culture media (Dugger et al., 2018; Holohan et al., 2013). This complication may be one of the explanations for the poor translation of many promising therapies to clinical application.

To overcome this problem, we recently developed a nerve‐muscle co‐culture system that resulted in the development of functional NMJ and highly differentiated contracting myotubes, without the need of serum or growth factors in the medium (Saini et al., 2019, 2020; Abd Al Samid et al., 2018). This suggests that all the factors required for the formation and maturation of NMJ and advanced differentiation of skeletal muscle cells were secreted endogenously. We, therefore, hypothesized that motor neurons and skeletal muscle cells in this co‐culture platform release all the necessary factors to stimulate myotube differentiation, sprouting of MN axons, and formation of functional NMJs. To investigate this, we applied immunohistochemistry to confirm the formation of NMJs, and ELISA‐based microarrays on supernatants collected form a‐neurally cultured human myoblasts and myoblasts co‐cultured with rat‐embryo spinal‐cord explants to examine the concentration of endogenously secreted growth factors and neurotrophins.

## 2 MATERIAL AND METHODS

### 2.1 Human myoblasts cell culture

Immortalized human myoblasts were cultured as described previously (Saini et al., 2019). Briefly, cells were seeded on 6‐well plates coated with 0.2% gelatin at a density of 150 × 10^3^ cells/mL. After 24 h, when the myoblast density reached ~90% confluency, they were washed twice with DPBS and incubated for 24 h at 37°C with 5% CO_2_ in differentiation medium (DM), consisting of 99% (v/v) DMEM, 1% (v/v) L‐glutamine, 10 µg/mL recombinant human insulin, and 10 µg/mL gentamicin, before plating the rat‐embryo spinal‐cord explants.

### 2.2 Isolation of rat embryonic spinal cord explants

All animal work undertaken was approved by the Home Office. Isolation of rat‐embryo spinal‐cord explants was carried out as described previously (Saini et al., 2019). The uterine horn was removed from the pregnant rat and embryo dissection was performed in a 100‐mm dish under a binocular microscope using 21‐gauge needles. The rat‐embryo spinal‐cord explant was dissected in one piece from each embryo and the surrounding connective tissue was removed, ensuring the dorsal root ganglia remained intact and attached to the rat‐embryo spinal‐cord explant, which was cut transversally into ~1–2 mm^3^ explants.

### 2.3 Co‐culture NMJ model and Immunocytochemistry

The co‐culture NMJ model was established and characterized by immunohistochemistry as described previously (Saini et al., 2019, 2020). Briefly, the DM was removed from the 6‐well plates and the cells were washed twice with DPBS. Then, 700 µL DM was added to each well. Three to six evenly spaced explants were placed into each well and incubated for 6 h to allow the explants to adhere with the SkMCs. Following the 6‐h incubation, additional DM was added dropwise to each dish to prevent dehydration of the skeletal muscle cells and rat‐embryo spinal‐cord explants. The cells were then incubated for an additional 24 h before adding further DM to each well. Between 24 and 48 h, the myocytes fuse into immature myotubes and sprouting neurites from the explants innervate the cells. Co‐cultures were maintained by changing half the DM every 48 h. Live cells were visualized using a Leica DMI6000 B inverted microscope from Leica Microsystems. Functional NMJ model was characterized using the primary antibodies (Anti‐Choline Acetyltransferase (ChAT) from Merck Millipore 1:100; α‐Bungarotoxin, Alexa Fluor® 647 conjugate 1:400; Myosin 4 monoclonal antibody (MF20), Alexa Fluor® 488‐anti‐MHC 1:500) that were added to the cells and incubated for 18–24 h at 4°C. Confirmation of myotube innervation and NMJ formation was assessed via confocal and immunofluorescence microscopy using a Leica DMI6000 B inverted microscope and a Leica TCS SP5 confocal microscope.

### 2.4 ELISA‐based microarray

In parallel, experiments were conducted to compare the concentration of 40 human growth factors in aneural myotube cultures and co‐cultured myotubes. One ELISA‐based microarray standard glass slide is spotted with 16 wells of identical growth factor antibody arrays (Human Growth Factor Antibody, Cambridge Bioscience). Each well was spotted with different controls: positive control spot (a control for the amount of biotinylated antibody printed onto the array), negative control spot (buffer printed, to measure the baseline responses) and a blank spot (nothing printed, to measure the background response). Each antibody was arrayed in quadruplicate. On day 7, when spontaneous myotube contractions in unison as a motor unit were first observed, supernatants were collected from both conditions using a human growth factor array kit, as described by the manufacturer. The total protein concentration of each sample was determined with a protein assay kit (Pierce™ BCA Protein Assay Kit, Thermo Fisher) to ensure loading equal amounts of protein. The array spot signal densities were quantified with a GenePix 4000B laser scanner at 532 nm wavelength. Raw data from the visualized array images was generated and processed with the GenePix Pro 4.1 Microarray Acquisition & Analysis Software, further statistical analysis of the data was performed with the RayBiotech Q‐Analyzer® tool Software for QAH‐GF‐1.

### 2.5 Statistical analyses

The results presented are representative of a minimum of three independent experiments. Results were analyzed using SPSS 25.0 statistical analysis software with an independent 2‐sided t‐test. Normality of the data was tested with the Shapiro–Wilk test, and if data were not normally distributed they were log‐transformed before applying the t‐test. Data were expressed as mean plus/minus standard deviation (±SD). Statistical differences were analysed with unpaired t test. Statistical significance was accepted *if p* < 0.05.

## 3 RESULTS

### 3.1 Co‐culture and NMJ formation

**FIGURE 1:**
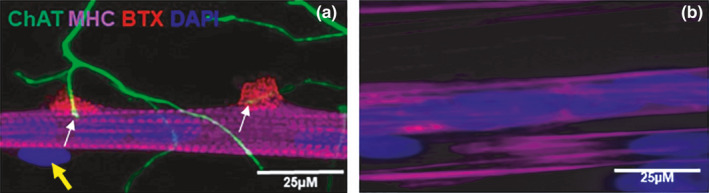
Image of neuromuscular junctions (NMJ) in the co‐culture of immortalized human skeletal muscle cells with rat‐embryo spinal‐cord explants (a) and aneurally cultured immortalized human skeletal muscle cells (b) at day 7. The panel A shows a representative image of a co‐culture stained for choline acetyltransferase (ChAT, green), α‐BTX (red), myosin heavy chain (MHC, magenta), and DAPI (blue). (a) white arrows indicate the terminal of motor neurons end at α‐BTX confirming the formation of NMJ. Yellow arrow indicates peripherally located nucleus. Scale bar = 25 µm.

To study the cross‐talk between muscle and motor neurons, we used our previously established co‐culture NMJ model (Saini et al., 2019). At day 7 of this co‐culture platform, the rat‐embryo spinal‐cord explants sprouted cholinergic motor neurons that branched to form multiple neuromuscular innervation site (Figure 1a). At the same time, the immortalized human myoblasts differentiated into striated myotubes with peripherally located nuclei and expressed acetylcholine receptors, as reflected by the α‐BTX stain as red clusters, confirming NMJ formation (Figure 1a). Aneurally cultured myoblast, on the other hand, did not show such a high degree of differentiation, as reflected by the absence of cross‐striation and peripherally located nuclei (Figure 1b).

**FIGURE 2:**
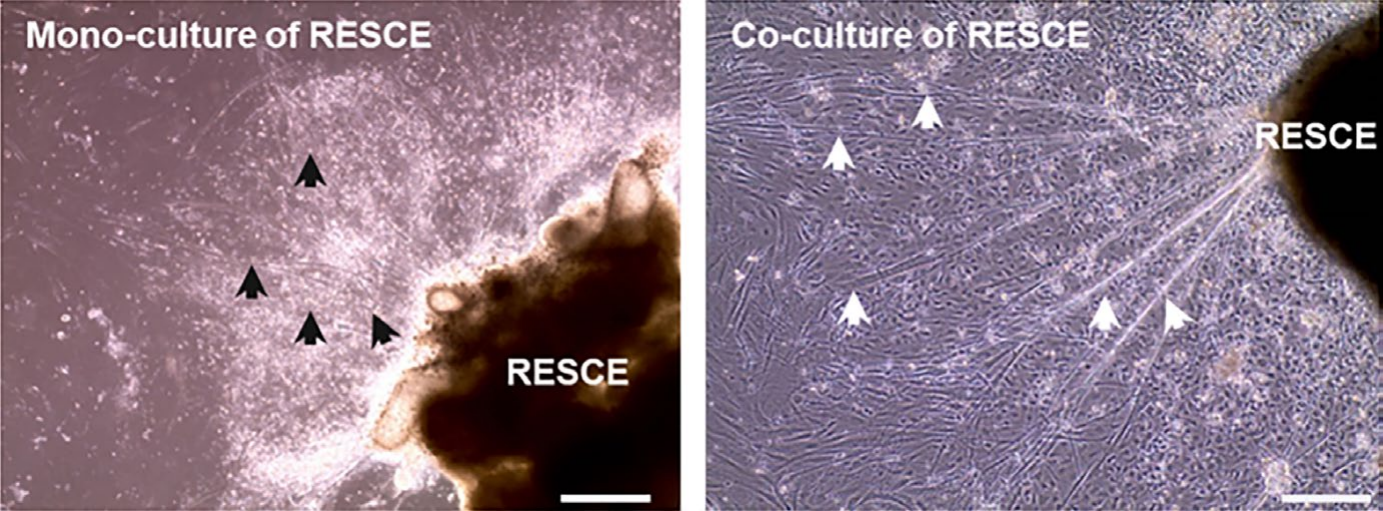
A representative image of mono‐ and co‐culture of RESCE at 72 h. Left panel: shows the deterioration of the mono‐culture of rat‐embryo spinal‐cord explants with small and short axons indicated by the black arrows. Right panel: demonstrates intact co‐culture of rat‐embryo spinal‐cord explants with large long axons indicated by white arrows. Scale bar =50 µm.

We also cultured just rat‐embryo spinal‐cord explants, to collect supernatant but they typically deteriorated and detached form the surface of the culture plate, despite it exhibited some growth with thin short neural outgrowth (Figure 2, left panel) within 72 h. At 7 day, rat‐embryo spinal‐cord explants were completely deformed and nothing was observed under the microscope. In contrast, rat‐embryo spinal‐cord explants co‐culture was intact at 72 h (Figure 2 right panel) sprouted long large axons compared with rat‐embryo spinal‐cord explants mono‐culture.

### 3.2 Quantification of growth and neurotrophic factors

To examine whether the co‐culture of myoblasts with rat‐embryo spinal‐cord explants enhances the endogenous secretion of the essential neural growth factor required for the formation of functional NMJs, an ELISA‐based microarray was performed on supernatant collected from the two culture conditions (co‐culture and a‐neural muscle). Among 40 growth/neurotrophic factors were quantified on day 7, seven factors were significantly more abundant in the co‐culture than in the a‐neurally cultured myotubes (*p* < 0.05).

**FIGURE 3:**
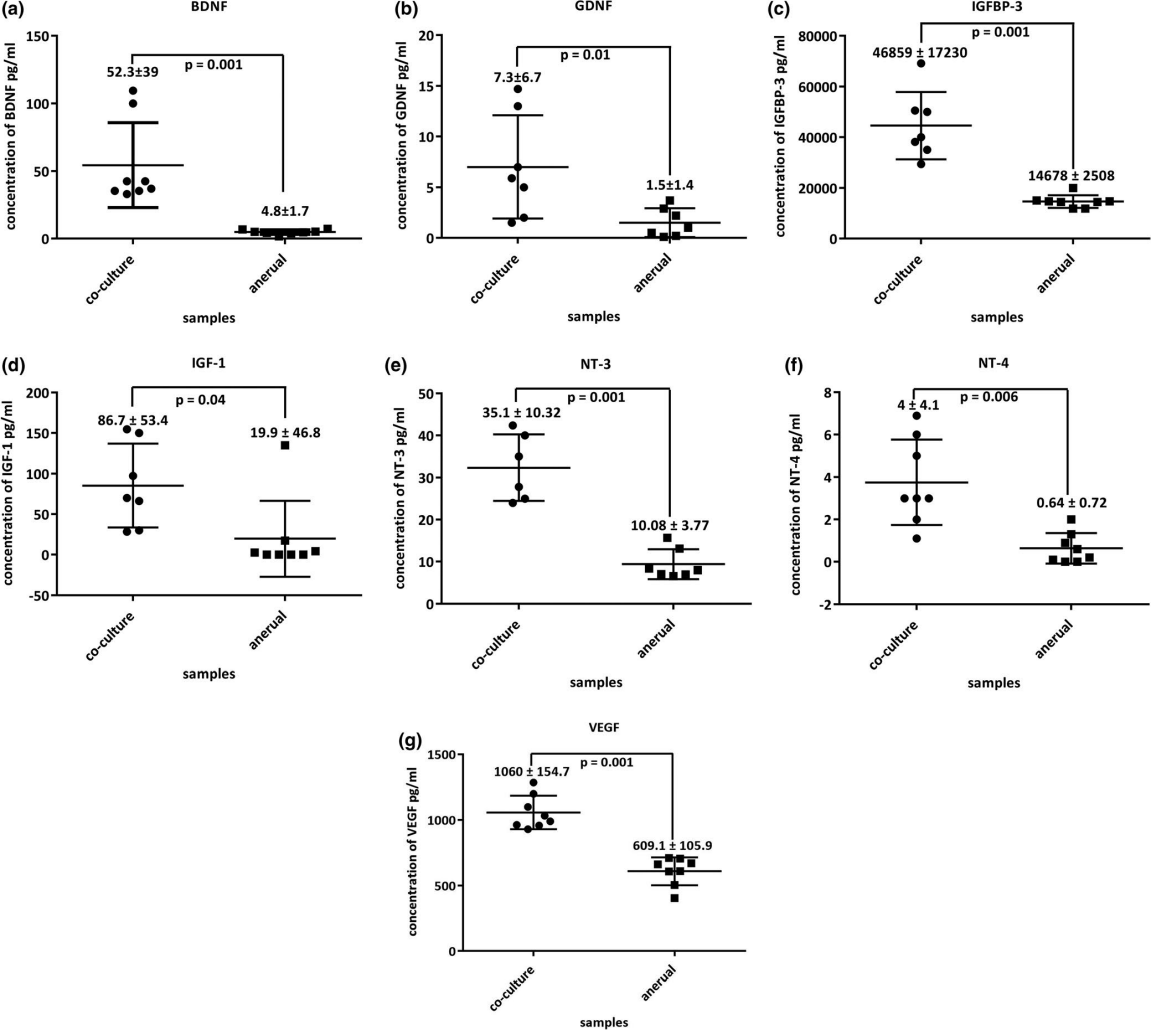
Scatter graphs for trophic factor quantified by ELISA‐based microarray of (a) BNDF, (b) GDNF, (c) IGFBP‐3, (d) IGF‐1, (e) NT‐3, (f) NT‐4, (g) VEGF. Data are presented as mean ± standard deviation. Each data point is the average of three independent experiments, *p* < 0.05 were considered statistically significant.

The concentrations of brain‐derived neurotrophic factor (BDNF, *p* < 0.001, Figure 3a), glial cell‐derived neurotrophic factor (GDNF, *p* = 0.01, Figure 3b), insulin‐like growth factor‐binding protein (IGFBP‐3, *p* < 0.001, Figure 3c) insulin‐like growth factor 1 (IGF‐1, *p* = 0.043, Figure 3d), neurotrophin (NT‐3, *p* < 0.001, Figure 3f) neurotrophin −4 (NT‐4, *p* = 0.006, Figure 3g), and vascular endothelial growth factor (VEGF, *p* < 0.001, Figure 3h) were higher in the supernatant collected from the co‐cultures compared to the supernatant from a‐neurally cultured myotubes, respectively.

## 4 DISCUSSION

Our co‐culture of human myoblasts and rat‐embryo spinal‐cord explants displays a high degree of myotube differentiation. Perhaps even more important is that the contraction of the myotubes in unison is realized through activation via functional NMJs as indicated by cessation of contractions by, for example, α‐bungarotoxin (Saini et al., 2020). As this functional innervation occurred without the addition of serum or growth factors to the system, it gave us the opportunity to assess the secretome of the system to obtain an indication of growth factors that most likely are essential for the formation of functional NMJs and muscle differentiation. Here we found that the abundance of BDNF, GDNF, NT‐3, NT‐4, VEGF, IGF‐1, and IGFBP‐3 were all higher in the co‐culture system than in a‐neurally cultured myoblasts. This suggests that the cross‐talk between the neural and muscle tissue enhances the secretion of growth factors important for muscle differentiation and NMJ formation and maintenance. The observation that a mono‐culture of rat‐embryo spinal‐cord explants did not grow for 7 days and usually deteriorate within 72 h further indicates that the cross‐talk is not only important for the maintenance and differentiation of the muscle cells, but also for the maintenance of the rat‐embryo spinal‐cord explants.

It has been reported that BDNF not only enhances the transmission at the NMJ, but also skeletal muscle innervation and motor neuron survival (Yan et al., 1993; Zhang & Poo, 2002). This corresponds with the observation that myoblasts transiently express high levels of BDNF during embryonic development *in vivo* during NMJ and skeletal muscle fiber maturation (Griesbeck et al., 1995). Perhaps somewhat paradoxically, chronic exposure to elevated BDNF levels inhibited synaptogenesis *in vitro* (Peng et al., 2003) and in combination these data suggest that the timing of elevated BDNF levels is important not only for the NMJ formation during early muscle development, but also to allow pruning of the multiple NMJs on a single fiber during early muscle development. In line with this, we found here a 10‐fold higher BDNF concentration in the co‐culture system with functional NMJs than in the a‐neurally cultured system without NMJs. These results provide evidence that the endogenously secreted BDNF in the present co‐culture system allowed for the physiological formation and development of NMJs, similar to *in vivo* NMJ formation.

Other nerve‐muscle co‐culture systems require the inclusion of exogenous BDNF (Das et al., ,,^2007^, ^2010^; Guo et al., ,^2011^, ^2014^, ^2017^; Puttonen et al., 2015; Rumsey et al., 2010; Smith et al., 2016; Vilmont et al., 2016) and yet do not generate the functional NMJ observed in our co‐culture system. This may well be a reflection of unsuitable concentrations of exogenous BDNF inhibiting NMJ maturation (Song & Jin, 2015) and/or illustrate the importance of the temporal variations in BDNF concentrations for NMJ formation as seen during *in vivo* NMJ formation. It is thus of interest to investigate the time course of the changes in BDNF concentrations in our co‐culture system during the development of NMJs and to what extent these changes in BDNF concentration are associated with multiple innervation.

We also observed elevated GDNF in the co‐cultures in comparison to myotube monocultures. GDNF is a key factor for motor neuron survival *in vitro* (Oppenheim et al., 1995). Intriguingly, GDNF is expressed by skeletal muscle while its receptor RET tyrosine kinase is expressed in the motor neurons (Baudet et al., 2008), a design illustrating the cross‐talk between the skeletal muscle and motor neurons. The significance of this receptor became apparent as obvious disruptions to motor neuron maturation and reduced MEP size at NMJs when conditionally ablating RET tyrosine kinase in the cranial motor neurons of mice (Baudet et al., 2008). In addition, a frog nerve‐muscle co‐culture system demonstrated that treatment with GDNF led to an increase in spontaneous synaptic current frequency and amplitude, further indicating its possible role as a retrograde signalling factor (Wang et al., 2002). Furthermore, studies with transgenic mice that overexpress skeletal muscle‐derived GDNF showed hyperinnervation of NMJs (Nguyen et al., 1998), motor unit enlargement and slowed synapse elimination (Keller‐Peck et al., 2001). These findings suggest skeletal muscle‐derived GDNF plays a role in the regulation of maturation of the NMJ and that the elevated GDNF in our co‐culture system enhances the differentiation of pre‐ and post‐synaptic components of NMJs in the system. Given the hyperinnervation with continuous GDNF stimulation (21, 45), it would be of interest to assess the time course of the GDNF levels, and how it is related to the maturational status and potential multiple innervation in our system.

IGF‐1 is a potent anabolic hormone that has been shown to induce hypertrophy in skeletal muscle in animal models and muscle cell culture systems (Velloso, 2008). The muscle specific IGF‐1 also plays a role in stabilizing NMJs and enhanced motor neuronal survival (Dobrowolny et al., 2005) and when injected directly into skeletal muscle of mice, IGF‐1 inhibits degeneration of motor neurons and NMJs, and the associated age‐related decline in force generating capacity (Payne et al., 2006). The IGF‐1 bioavailability is enhanced through the activity of the IGFBPs (Stewart et al., 1993). IGFBP‐3 is the most common protein of the IGFBP superfamily (Adachi et al., 2017), acts as a membrane transporter protein for IGF‐1 (Hwa et al., 1999) and binds 80% of the circulating IGF‐1 with a 1:1 ratio. Noteworthy, IGFBP‐3 was also the most abundant IGFBP in both the co‐cultures and monocultures. In addition to IGF‐1, also IGFBP‐3 expression was elevated in the co‐cultures and further indicates the significance of the cross‐talk between rat‐embryo spinal‐cord explants and skeletal muscle cells in advanced differentiation of myofibers, and the formation of robust NMJ, comparable to the development *in vivo*.

NT‐3 and NT‐4 were also elevated in the co‐cultures when compared to a‐neural myotube cultures. Studies have demonstrated that NT‐3 and NT‐4 are important modulators of synaptic function and development, and are required for maintenance of presynaptic and postsynaptic structures at the NMJ (Belluardo et al., 2001; Gonzalez et al., 1999). In addition, NT‐3 plays an important role in motor neuron differentiation as reflected by the smaller motor neuron soma size during early postnatal development of NT‐3‐deficient mice, even though the number of α‐motor neurons was not significantly affected (Woolley et al., 1999). This smaller motor neuron size in NT‐3‐deficient mice was accompanied by a decreased number of motor end plates (MEPs) and a lower number of skeletal muscle fibers at birth, followed by a catastrophic postnatal loss of motor neurons and complete denervation of hindlimb muscles with no observable NMJ remaining (Woolley et al., 2005). Similar observations were made in mice with haploinsufficiency‐induced reductions in NT‐3 (Sheard et al., 2010) and indicate the importance of NT‐3 in NMJ formation, and muscle development and maintenance. Another neurotrophin, NT‐4, has been shown to enhance NMJ transmission in a phrenic nerve / adult rat diaphragm‐muscle system during transmission failure induced by repetitive nerve stimulation (Mantilla et al., 2004). Interestingly, the expression of NT‐4 is dependent on synapse activity at the NMJ, as blockade of AChRs on the NMJ MEP with α‐BTX caused a reduced NT‐4 expression, while electrical stimulation of motor neurons enhances skeletal muscle‐derived NT‐4 expression (Funakoshi et al., 1995). The elevated expression of NT‐3 and NT‐4 in our co‐culture system may thus well have contributed to NMJ formation, development and synaptic activity.

The concentration of VEGF, a member of the VEGF sub‐family, was also significantly elevated in the co‐cultures when compared to the a‐neural myotube cultures. Originally described for their angiogenic role (Ferrara, 2004), the VEGF family of factors has also important functions in motor neuron growth, guidance, migration, and survival (Rosenstein et al., 2010; Ruiz de Almodovar et al., 2009). Indeed, in amyotrophic lateral sclerosis transgenic mice systemic administration of VEGF resulted in an increase in the number of NMJs in the diseased mice (Zheng et al., 2007). Furthermore, administration of VEGF in mice with ischemic injury promoted both regrowth and maintenance of damaged motor neuron axons in the mice (Shvartsman et al., 2014) and was accompanied with an increased expression of GDNF and NGF that aid motor neuron axon regeneration (Shvartsman et al., 2014). Thus, the higher VEGF expression in the co‐culture than the a‐neurally cultured myoblasts may well have led to the elevated expression of GDNF in the co‐culture and the development of functional NMJs.

## 5 LIMITATION

It should be noted that our array was human‐specific and we had a rat‐human co‐culture. This may be considered a limitation, but growth factors are quite similar in rats and humans. Perhaps even more indicative that this is a minor problem was our ability to detect significant differences in the expression of growth factors between the aneurally cultured human myoblasts, and the rat‐embryo spinal‐cord explants and human myoblast co‐culture. It remains to be seen, however, whether the growth factors have a neural or muscular origin in this co‐culture system. Even if cross‐reactivity is a problem, our co‐culture system clearly showed the beneficial effects of cross‐talk between muscle and MN on NMJ formation that was related to elevated concentrations of growth factors in the medium that must have come from the nervous and/or muscle cells in the system.

## 6 CONCLUSION

In summary, this study shows that the formation of functional NMJs is the result of an orchestrated cross‐talk between motor neurons and muscle fibers through endogenously released trophic and growth factors. Furthermore, the results demonstrated the formation and development of NMJs as well as advanced differentiation of immortalized human myotubes *in vitro*, through the precise expression of essential growth factors and neurotrophins. It can not be excluded that other secreted proteins contribute to this bidirectional communication. Future studies using high‐throughput microarrays, such as antibody‐microarrays, may identify additional differentially expressed proteins in the secretome. Thus, this novel co‐culture system, free from serum, growth, and neurotrophic factors is an ideal *in vitro* NMJ model to screen potential drugs and molecules of interest to treat neuromuscular and neurodegenerative disorders.

## CONFLICTS OF INTEREST

The authors report no conflicts of interest in this work.

## AUTHOR CONTRIBUTIONS

J.S., A.F., A.J.R., H.D., and N.A.S. developed the study concept. All the authors (J.S.; A.F.; A.J.R.; K.M.; V.M.; G.B.‐B.; A.P.L.; J.S.M.; H.D.; N.A.S.) contributed to experimental procedures. J.S., A.F. and N.A.S. carried out the investigation. A.J.R., J.S.M., H.D., and N.A.S. supervised the study. N.A.S. and H.D. wrote the original draft of the manuscript. J.S., A.P. L., J.S.M., H.D., and N.A.S. reviewed and edited the draft manuscript. N.A.S. and H.D. performed the data analysis, drafted, and revised the article. All authors have done proof‐reading and gave final approval of the version to be published and agreed to be accountable for all aspects of the work.
